# Deep remission from induction chemotherapy predicts favorable long-term survivals in early stage extranodal nasal NK/T-cell lymphoma receiving sequential chemotherapy and radiation

**DOI:** 10.18632/aging.204355

**Published:** 2022-11-01

**Authors:** Fei Qi, Wenyuan Zhou, Yan Xie, Yan Sun, Meng Wu, Yue Chai, Bo Chen, Ningjing Lin, Weiping Liu, Ning Ding, Yexiong Li, Mei Dong, Yuqin Song, Jun Zhu

**Affiliations:** 1 Key Laboratory of Carcinogenesis and Translational Research (Ministry of Education/Beijing), Department of Lymphoma, Peking University Cancer Hospital and Institute, Beijing 100142, China; 2 NMPA Key Laboratory for Research and Evaluation of Radiopharmaceuticals (National Medical Products Administration), Department of Nuclear Medicine, Peking University Cancer Hospital and Institute, Beijing 100142, China; 3 Department of Radiation Oncology, Peking University Cancer Hospital and Institute, Beijing 100142, China; 4 Department of Medical Oncology, National Cancer Center/National Clinical Research Center for Cancer/Cancer Hospital, Chinese Academy of Medical Sciences and Peking Union Medical College, Beijing 100021, China; 5 Department of Radiation Oncology, National Cancer Center/National Clinical Research Center for Cancer/Cancer Hospital, Chinese Academy of Medical Sciences and Peking Union Medical College, Beijing 100021, China

**Keywords:** extranodal NK/T-cell lymphoma, induction chemotherapy, response, survival, individualized treatment

## Abstract

Objective: We aimed to assess the association between induction chemotherapy (CT) response and survivals and to explore an induction CT response-adapted treatment strategy for localized extranodal NK/T-cell lymphoma (NKTCL) receiving first-line sequential CT and radiation (RT).

Methods: We retrospectively reviewed the data of patients with localized NKTCL receiving first-line CT+RT from 2010 to 2020 at two independent institutes (primary cohort, *n* = 203; validation cohort, *n* = 67). Responses after induction CT (initial response), RT (final response) and survivals were analyzed.

Results: Patients with initial complete remission (CR) had higher final CR rate than the others (99.1% vs. 78.7%, *P* < 0.001). Initial CR was associated with superior 5-year progression-free survival (PFS, 90.0% vs. 61.4% vs. 30.8%, *P* < 0.001) and overall survival (OS, 93.5% vs. 70.7% vs. 60.6%, *P* < 0.001), as compared to initial partial remission or non-response. Though majority of cases with initial non-CR achieved final CR after RT, they still had a tendency of shortened OS compared with initial CRs (86.9% vs. 90.6%, *P* = 0.063). Multivariate analysis demonstrated patients with initial non-CR had higher relapse (HR = 4.748, 95% CI, 2.396–9.407, *P* < 0.001) and death hazard (HR = 4.296, 95% CI, 1.802–10.24, *P* = 0.001). Furthermore, more intensive therapy of ≥6 total cycles of CT yielded significantly superior 5-year OS for patients with initial non-CR (76.7% vs. 54.7%, *P* = 0.026) rather than patients with initial CR.

Conclusion: Deep remission from induction CT was associated with favorable survivals in localized NKTCL receiving CT+RT, and an induction CT response-adapted individualized treatment strategy might be recommended in clinical practice.

## INTRODUCTION

Extranodal NK/T-cell lymphoma, nasal type (NKTCL) is a subtype of mature NK or T cell lymphoma characterized by extranodal involvement and association with Epstein-Barr virus [1–3]. It has a geographical predilection for Asian and Latin American populations [4, 5]. Majority (70–80%) of cases present localized disease predominately occurring in the upper aerodigestive tract (UADT), especially nasal cavity and Waldeyer’s ring [1, 6]. Tumor cells exhibit a cytotoxic phenotype, primarily characterized by the expression of perforin and granzyme B [1].

NKTCL is unique among common aggressive lymphomas in terms of treatment principles. It is highly sensitive to radiation (RT) but resistant to chemotherapy (CT), especially anthracycline (ANT)-based regimens [7–9]. RT is the backbone for the treatment of localized NKTCL and is the most effective modality to make rapid locoregional disease control (LRC) [8, 10–12]. RT alone achieved excellent complete remission (CR) rate of 83–100% and satisfactory 5-year overall survival (OS) rate of 83.3–88.8% for stage I diseases [8, 13, 14]. Besides, the addition of CT to RT brings further survival benefits, especially among high-risk cases [7, 14, 15]. Currently, the combined chemoradiation (CRT), arranged sequentially or concurrently, is regarded as a standard treatment modality for early stage NKTCL.

In clinical practice, first-line sequential induction CT followed by RT is widely used in treating early stage NKTCL. On one hand, the sequential modality achieves similar responses and survivals as concurrent CRT [16–24]. On the other, it is much easier to arrange without waiting for RT planning, and is also more tolerable since it avoids intensifying mucositis and overlapping of other toxicities [25]. In the modality of sequential CT and RT, though non-ANT-based regimens yielded satisfactory ORR of 81–92% after induction CT, the remission was not deep with only half or less cases reaching CR [17, 26–29]. Fortunately, RT still produced excellent LRC among those non-CRs, and dramatically improved the CR rate to 70–100% at the completion of RT [26, 29–31]. Therefore, in the setting of effective RT, is it still necessary to achieve early deep remission from induction CT? Up to now, however, the association between induction CT response and survival of localized NKTCL has not been fully explored.

Optimization of risk stratification is vital for clinical decision guidance in localized NKTCL since 10–37% of cases still have refractory disease or progression after first-line CRT [32]. Li and his colleagues reviewed 214 patients with early stage NKTCL, and results revealed that systemic dissemination was the main failure pattern [32]. The 5-year cumulative incidences of systemic failure (SF) were similar between patients receiving RT alone and CRT (28.5% vs. 24.1%, *P* = 0.758) [32]. Therefore, it is inferred that more effective systemic therapy is one of the key elements for further cure of this disease. At present, an early recognition and selection of vulnerable patients for intensive therapy are warranted. Apart from well-established pretreatment prognostic factors [33–37], treatment-related variables reflecting tumor reaction to therapy, such as post-treatment EBV-DNA positivity, positron emission tomography (PET) scan after primary treatment, and inadequate dose of RT were reported to predict early relapse and poor prognosis for localized NKTCL [12, 38, 39]. Here, we suppose that the induction CT response, which reflects tumor’s sensitivity to systemic therapy, might also be a potential prognostic factor for long-term survivals and might be used in individualized treatment for localized NKTCL.

Therefore, among stage I(E)/II(E) NKTCL patients who received first-line sequential CT and RT, we conducted this retrospective study to assess the association between induction CT response and long-term survivals; we also analyzed the failure patterns and further explored its potential role in guiding treatment strategy for NKTCL.

## RESULTS

### Patient population description

Patients who progressed rapidly during or after induction CT with no chance for subsequent RT were excluded. Finally, a total of 203 consecutive patients who were newly-diagnosed localized NKTCL and underwent first-line CT and RT from 2010 to 2020 from Peking University Cancer Hospital were retrospectively reviewed as the primary cohort; another independent group of 67 patients from Cancer Hospital, Chinese Academy of Medical Sciences and Peking Union Medical College were analyzed as the validation cohort. Baseline patient characteristics were summarized in Table 1. For patients in the primary cohort, the median age was 42 (range, 15–83) years, and the male-to-female ratio was 2.12:1. All patients had good performance status score of 0–1. Stage I disease accounted for 45.8%, and stage II accounted for 54.2% of patients. Elevated lactate dehydrogenase (LDH) was presented in 24.1% of cases, PTI in 53.7% of cases, and B symptoms in 38.9% of cases. Patients in the primary cohort presented a high-risk population with intermediate- and high-risk patients (NRI > 1) accounted for 76.4% according to NRI stratification. Baseline characteristics were well balanced between the validation and primary cohort except for the serum LDH, which was higher among the validation cohort of patients (40.3% vs. 24.1%, *P* = 0.018).

**Table 1:** Patient baseline characteristics.

**Characteristics**	**Primary cohort**	**Validation cohort**	
Gender			1.000
Male	138 (68.0)	46 (68.7)	
Female	65 (32.0)	21 (31.3)	
Age			0.646
≤60	183 (90.1)	59 (88.1)	
>60	20 (9.9)	8 (11.9)	
ECOG score			0.248
0–1	203 (100.0)	66 (98.5)	
≥2	0 (0)	1 (1.5)	
Primary site			0.617
Nasal cavity	158 (77.8)	50 (74.6)	
Extra-nasal	45 (22.2)	17 (25.4)	
Stage			1.000
I (E)	93 (45.8)	31 (46.3)	
II (E)	110 (54.2)	36 (53.7)	
B symptom			0.069
Absent	130 (59.1)	30 (44.8)	
Present	90 (40.9)	37 (55.2)	
Primary tumor invasion			0.779
No	94 (46.3)	33 (49.3)	
Yes	109 (53.7)	34 (50.7)	
Serum LDH			0.018
Normal	154 (75.9)	40 (59.7)	
Elevated	49 (24.1)	27 (40.3)	
IPI score			1.000
Low risk	187 (92.1)	60 (89.6)	
Intermediate-low risk	14 (6.9)	7 (10.4)	
Intermediate-high risk	1 (0.5)	0	
High risk	1 (0.5)	0	
PINK			0.254
0	184 (90.6)	57 (85.1)	
1	19 (9.4)	10 (14.9)	
NRI			0.154
Low risk	48 (23.6)	8 (11.9)	
Intermediate-low risk	52 (25.6)	17 (25.4)	
Intermediate-high risk	69 (34.0)	30 (44.8)	
High risk	34 (16.7)	12 (17.9)	

Abbreviations: ECOG: eastern cooperative oncology group performance status; LDH: lactate dehydrogenase; IPI: International Prognostic Index; PINK: Prognostic index of natural killer lymphoma; NRI: nomogram-revised risk index.

### Treatment responses

**Figure 1:**
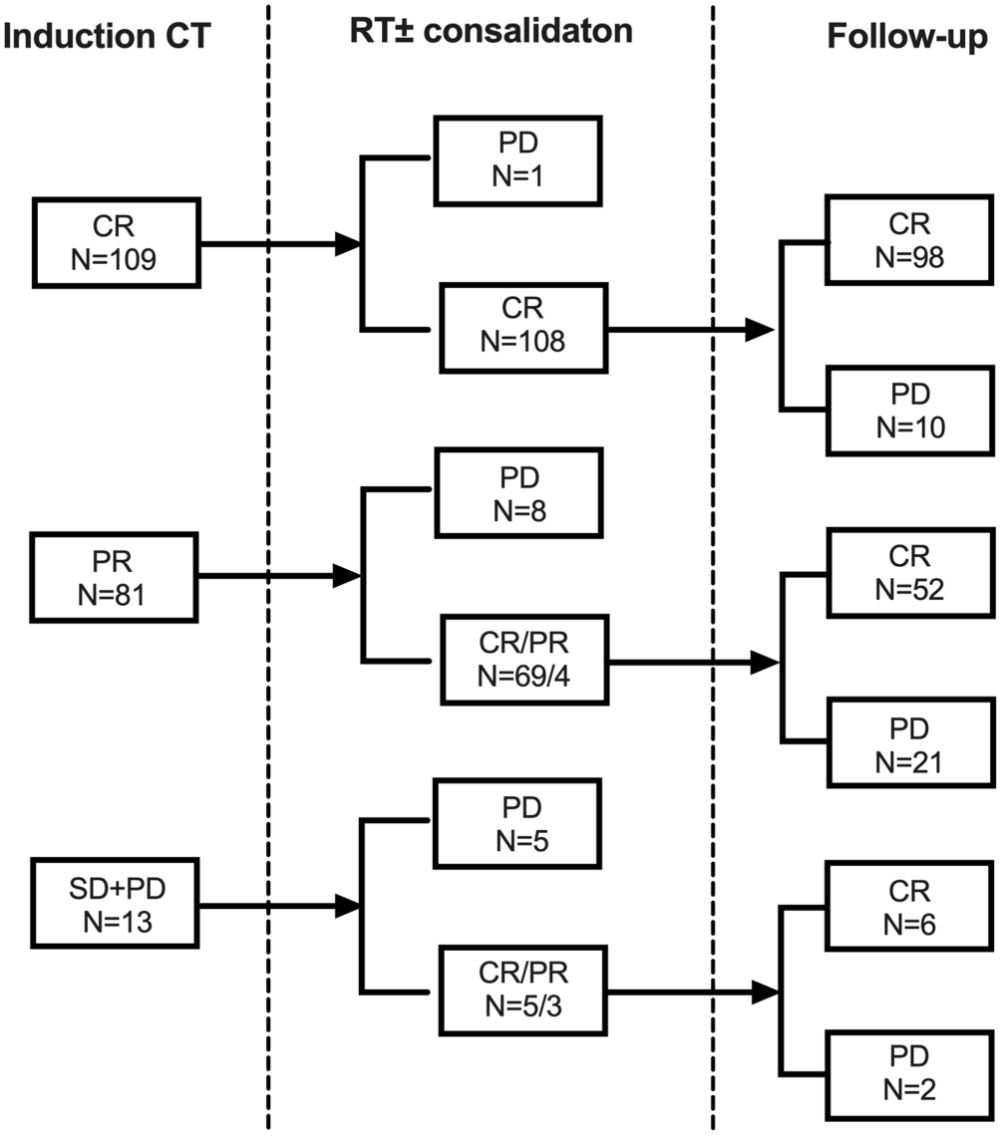
**Responses from induction chemotherapy and subsequent radiotherapy for early stage NKTCL in the primary cohort.** Abbreviations: CT: chemotherapy; RT: radiotherapy; CR: complete remission; PR: partial remission; SD: stable disease; PD: progression of disease.

Treatment response was shown in Figure 1. A total of 134 patients (66.0%) used PET scanning for induction response assessment. The initial CR rate after induction CT was 53.7%. All but one patient (99.1%) with initial CR maintained final CR after RT. Subsequent RT further deepened the remission: 69 out of 81 (85.2%) cases with initial partial remission (PR), and 5 out of 13 (38.5%) initial non-responders achieved final CR after RT. In conclusion, compared with initial non-CRs, patients who were highly sensitive to induction CT had higher final CR rate after subsequent RT (99.1% vs. 78.7%, *P* < 0.001). That was the same in the validation cohort: the final CR rate was 96.3% for patients with initial CR versus 77.8% for patients with initial non-CR (*P* < 0.001).

### Induction CT responses predicted survivals in localized NKTCL receiving CRT

**Figure 2:**
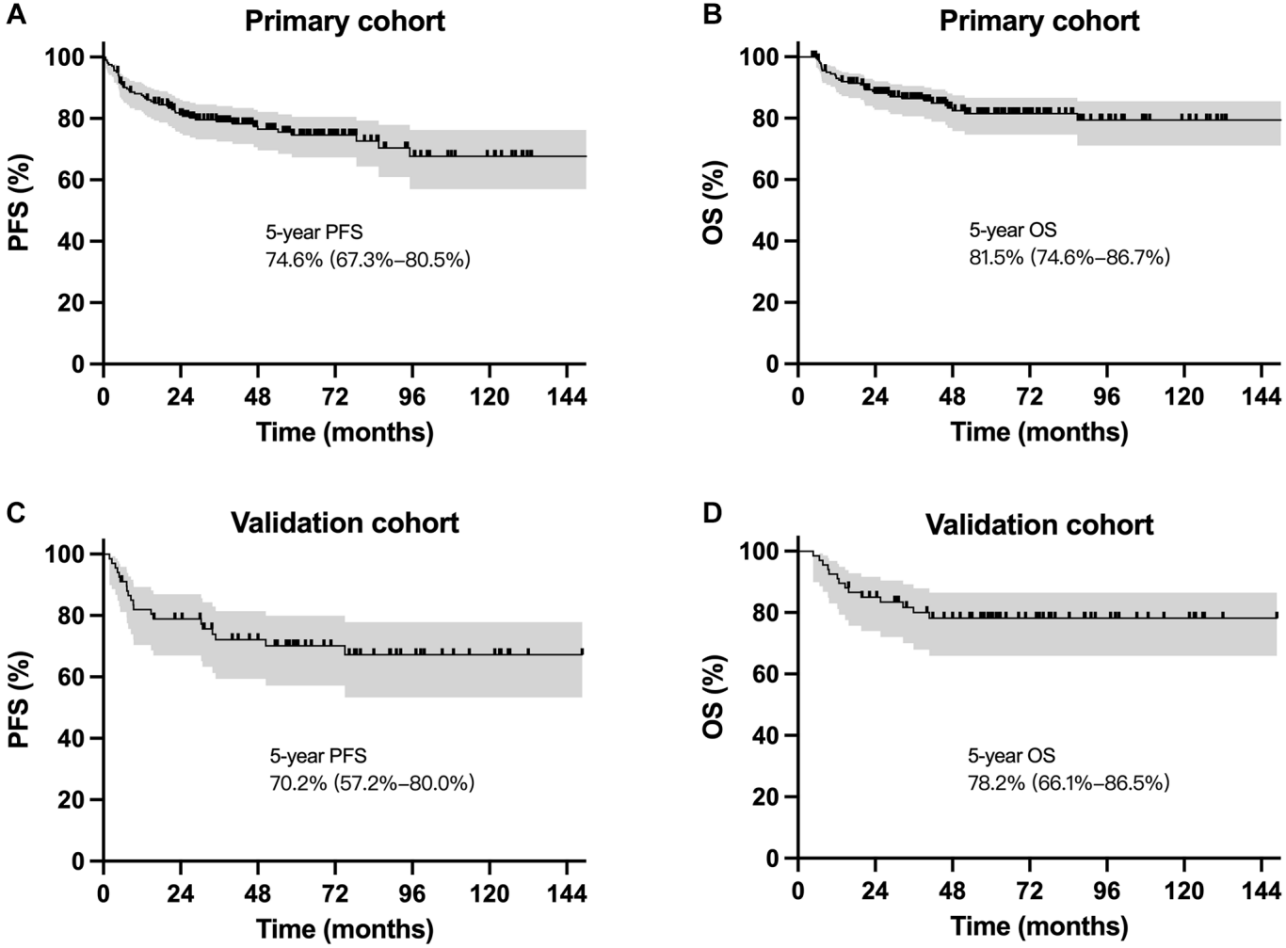
**Survival curves for patients with localized NKTCL receiving sequential chemoradiotherapy.** Progression-free survival and overall survival cures for the primary cohort (**A** and **B**) and the validation cohort (**C** and **D**).

Till the last visit in February 2022, the median follow-up period was 59.2 months for surviving patients in the primary cohort and 62.7 months in the validation cohort. The 5-year progression-free survival (PFS) and OS were 74.6% and 81.5%, respectively for the primary cohort, and 70.2% and 78.2%, respectively for the validation cohort (Figure 2). Survival outcomes were comparable for patients receiving different regimens in the primary cohort and the validation cohort, respectively.

**Figure 3:**
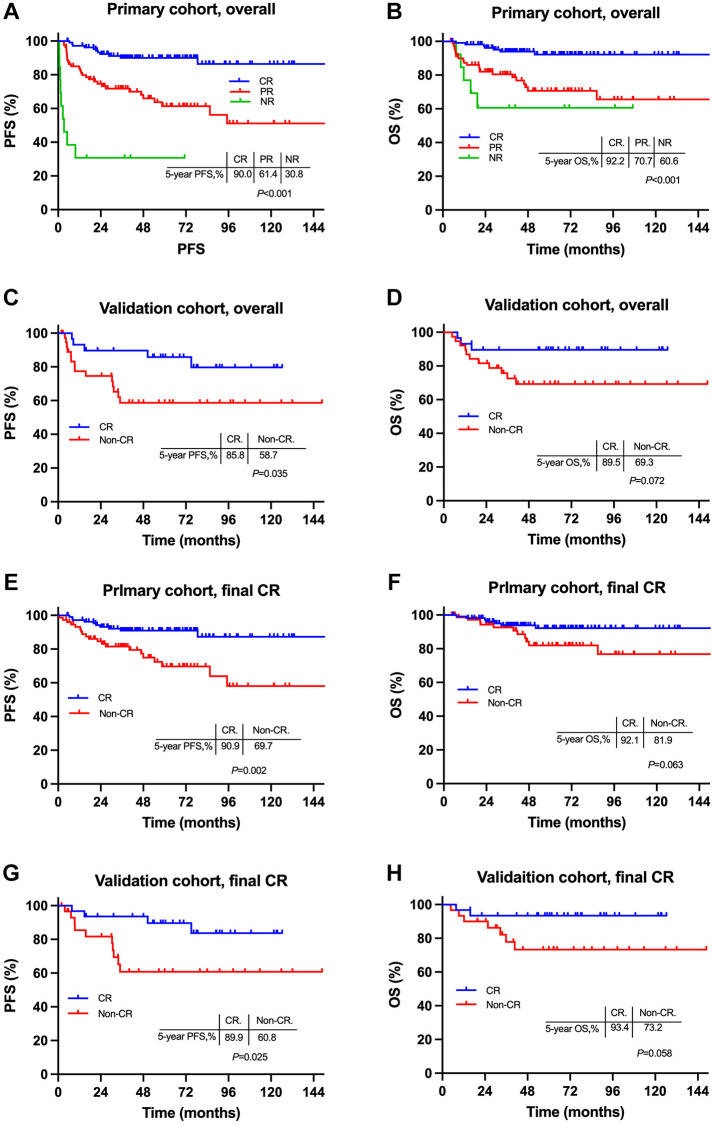
**Comparisons of survivals between early stage NKTCL patients with different induction chemotherapy responses.** Survival cures for entire patients in the primary cohort (**A** and **B**) and the validation cohort (**C** and **D**); survival cures for patients with final CR in the primary cohort (**E** and **F**) and the validation cohort (**G** and **H**). Abbreviations: PFS: progression-free survival; OS: overall survival; CR: complete remission; PR: partial remission; NR: non-response.

For patients with initial CR after induction CT in the primary cohort, the 5-year PFS and OS rates were 90.0% and 92.2%, respectively, much superior to those of initial PRs (61.4% and 70.7%, *P* < 0.001; Figure 3A and 3B) or non-responders (30.8% and 60.6%, *P* < 0.001; Figure 3A and 3B). Furthermore, among patients who had final CR after subsequent RT, those with initial CR still had superior 5-year PFS compared with initial non-CRs (90.9% vs. 69.7%, *P* = 0.002; Figure 3E); though the difference did not reach statistical significance, initial CR did present a tendency of prolonged OS over initial non-CRs (92.1% vs. 81.9%, *P* = 0.063; Figure 3F). Similar results were observed among patients in the validation cohort (Figure 3C, 3D, 3G and 3H).

Multivariate analysis demonstrated induction CT response as an independent predictor for PFS and OS (Table 2). It was no doubt that patients with initial non-responders (vs. initial CR) to induction CT had higher risk of developing relapse (HR = 19.90, 95% CI, 7.338–54.46; *P* < 0.001) and death (HR = 4.256, 95% CI, 1.239–14.62; *P* = 0.021). Noteworthy, those with initial PR also had significantly higher relapse hazard (HR = 3.934, 95% CI, 1.928–8.025; *P* < 0.001) and death hazard (HR = 4.801, 95% CI, 1.960–11.76, *P* = 0.001) in comparison to initial CRs.

**Table 2:** Multivariate analysis for survivals for patients in the primary cohort.

**Characteristics**	**PFS**		**OS**	
	**HR (95% CI)**	***P*-value**	**HR (95% CI)**	***P*-value**
Age, >60 y vs. ≤60 y	2.069 (0.847–5.057)	0.111	3.750 (1.449–9.705)	0.006
Primary site, extra-nasal vs. nasal	1.171 (0.601–2.282)	0.643	1.172 (0.603–2.279)	0.639
Stage, II (E) vs. I (E)	3.141 (1.512–6.528)	0.002	3.053(1.472–6.335)	0.003
B symptom, yes vs. no	1.035 (0.554–1.933)	0.913	1.182 (0.648–2.154)	0.586
LDH, elevated vs. normal	1.703 (0.909–3.191)	0.096	1.186 (0.550–2.555)	0.664
PTI, yes vs. no	1.228 (0.648–2.326)	0.529	2.214 (0.977–5.017)	0.057
Induction response				
PR vs. CR	3.934 (1.928–8.025)	<0.001	4.801 (1.960–11.76)	0.001
Non-response vs. CR	19.90 (7.338–54.46)	<0.001	4.256 (1.239–14.62)	0.021

Abbreviations: CR: complete remission; PR: partial remission; LDH: lactate dehydrogenase; PTI: primary tumor invasion; PFS: progression-free survival; OS: overall survival; HR: hazard ratio; 95% CI: 95% confidence interval.

### SF was the main failure pattern for localized NKTCL

**Figure 4:**
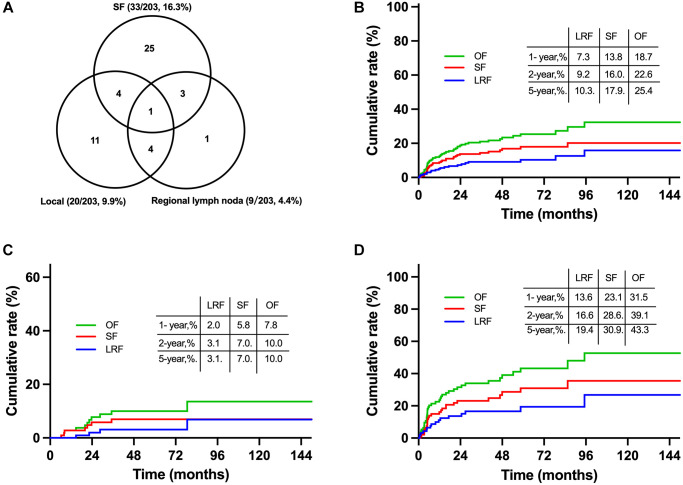
**Failure patterns and cumulative failure incidences for early stage NKTCL receiving combined chemoradiation in the primary cohort.** Failure patterns for entire patients in the primary cohort (**A**), cumulative failure rates for entire patients (**B**), patients with initial CR (**C**) and non-CR (**D**) from induction chemotherapy. Abbreviations: OF: overall failure; SF: systemic failure; LRF: locoregional failure; CR: complete remission.

Till the last follow up, a total of 49 (24.1%) patients failed the treatment in the primary cohort including 24 (11.8%) cases with locoregional failure (LRF) and 33 (16.3%) cases with SF. Failure patterns were summarized in Figure 4A. Most failures occurred within the first two years after diagnosis. The 5-year cumulative incidences of overall failure (OF), LRF and SF were 25.4%, 10.3% and 17.9%, respectively (Figure 4B). Patients with initial CR had much lower OF, LRF and SF rates of 10.0%, 3.1% and 7.0%, respectively (Figure 4C). And the corresponding rates were 43.3%, 19.4%, and 30.9%, respectively, for patients with initial non-CR (Figure 4D). SF was the main failure pattern for localized NKTCL regardless of induction CT response, indicating the necessity of more intensive and effective systemic therapy.

### Induction CT response-adapted individualized therapy for localized NKTCL

**Figure 5:**
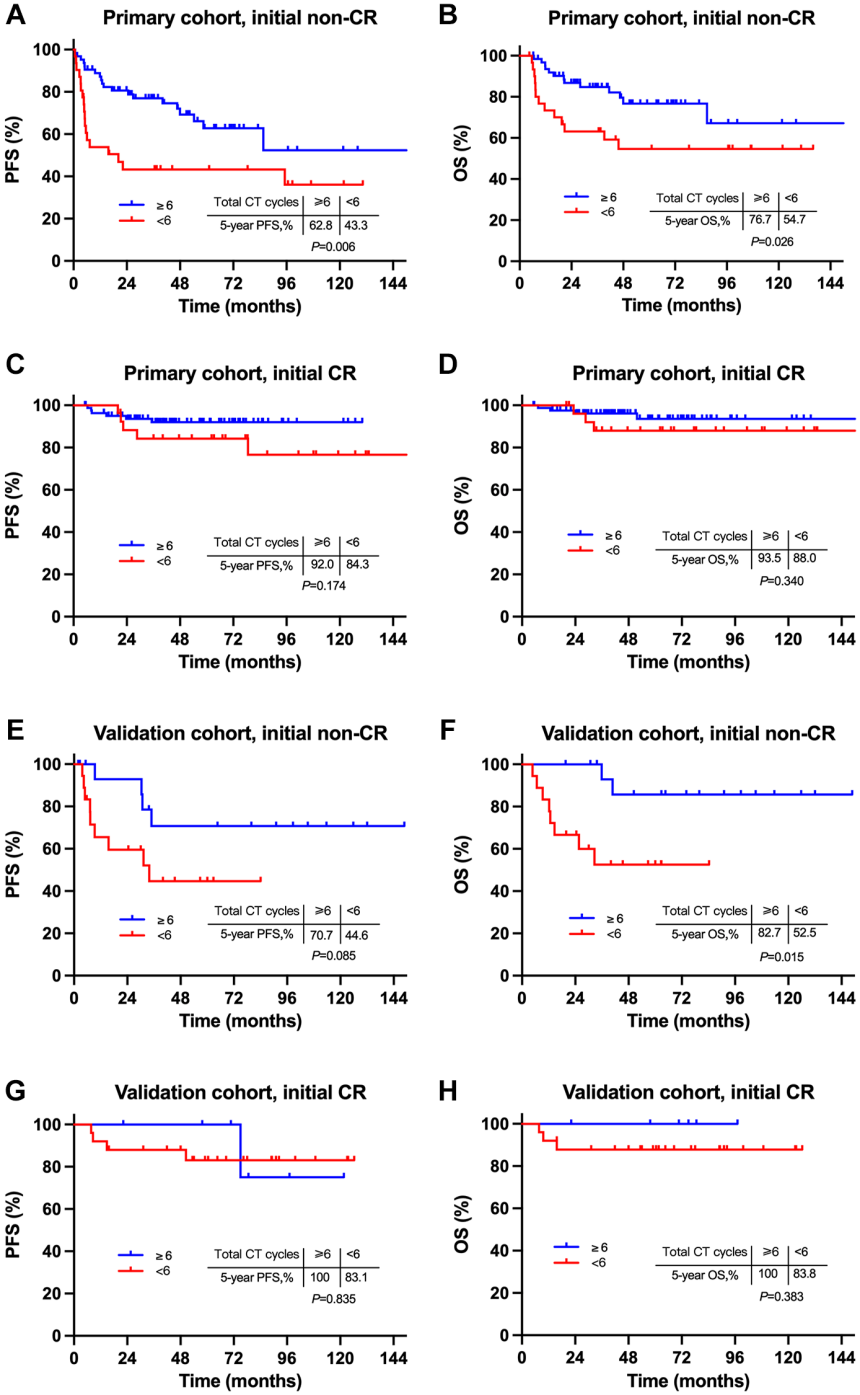
**Comparison of survivals between intensive and non-intensive chemotherapy for patients with different induction response.** Comparison of survivals between ≥6 cycles and <6 cycles of CT for patients with initial non-CR (**A** and **B**) and initial CR (**C** and **D**) in the primary cohort, initial non-CR (**E** and **F**) and initial CR (**G** and **H**) in the validation cohort. Abbreviations: PFS: progression-free survival; OS: overall survival; CR: complete remission.

We subsequently explored the influence of intensive systemic therapy on survivals among patients with different induction responses. For patients with initial PR after induction CT, those who had ≥ 6 total cycles of CT had significantly superior survivals to those who had < 6 total cycles of CT (5-year PFS, 62.8% vs. 43.3%, *P* = 0.006; 5-year OS, 76.7% vs. 54.7%, *P* = 0.026; Figure 5A and 5B). However, for those with initial CR after induction CT, survivals were comparable between a total ≥6 and <6 cycles of CT (5-year PFS, 92.0% vs. 84.3%, *P* = 0.174; 5-year OS, 93.5% vs. 88.0%, *P* = 0.340; Figure 5C and 5D). Similar results were observed in the validation cohort (Figure 5E–5H). These results revealed that the more intensive systemic therapy improved survivals of patients with initial non-CR whose main failure pattern was high rate of SF. Based on these results, we suggested an individualized treatment strategy based on induction CT response for localized NKTCL: more intensive therapy of ≥6 total cycles of CT be recommended to those who did not reached CR from induction CT, rather than those who had initial CR. This induction response-adapted strategy needs to be further validated by large-sample prospective studies since death event was few in some certain groups in our analysis.

## DISCUSSION

This is a comparably large-scale, real-world study which focuses on the association between first-line induction CT response and long-term survivals among early stage UADT-NKTCL. Firstly, our results revealed that early deep remission from induction CT was associated with prolonged PFS and OS in the presence of combined RT for localized NKTCL. Though most patients with initial non-CR got final CR after subsequent RT, they still had significantly higher relapse and death hazard than those with initial CR. Secondly, we demonstrated SF as the main failure pattern for patients receiving CRT, and those with initial non-CR had significantly higher cumulative incidence of SF. Lastly and importantly, we suggested an induction CT response-adapted individualized therapy for localized NKTCL that more intensive therapy of ≥6 total cycles of CT might be recommended for those with initial non-CR rather than initial CR from induction CT.

It is well established that RT is the most effective treatment modality for rapid LRC in localized NKTCL [8, 14, 29]. The CR rate of RT alone reached 70–100% for localized diseases, significantly higher than that of CT alone (25–50%) [8, 13, 40]. Improved LRC was revealed to translate into survival benefits: an absolute gain of 10% LRC provided a 9.54% improvement in PFS and 8.79% improvement in OS [10]. Meanwhile, the survival benefit of adding CT to RT has also been proved by several comparison studies [14, 15, 41]. In a retrospective analysis of 1360 early stage NKTCL patients from 20 institutions in China, combined CT+RT (vs. RT alone) significantly improved PFS (63.5% vs. 54.2%, *P* < 0.001) and OS (73.2% vs. 60.9%, *P* < 0.001) for intermediate-/high-risk NKTCL [15]. Moreover, the addition of new-regimens to RT achieved more survival benefit compared with ANT-based regimens (5-year OS, 59.5% vs. 44.5%) [41]. However, the association between tumor’s sensitivity to CT and survivals has not been fully discussed in localized NKTCL.

Our results proved induction CT response as an independent prognostic factor in NKTCL. In line with published studies, the CR rate was 53.7% after induction CT and improved to 89.7% after RT in our study [16, 17, 20–23, 26]. Patients who were highly sensitive to induction CT had higher final CR rate at the completion of RT (99.1% vs. 85.2%, *P* < 0.001), which might explain the survival advantage of patients with initial CR over initial non-CR from induction CT (5-year OS, 92.2% for initial CR vs. 70.7% for initial PR vs. 60.6% for initial non-response, *P* < 0.001). Furthermore, among patients who had final CR after RT, those with initial CR showed a tendency of prolonged OS, though the difference was not statistically significant. This indicated that RT was vital in improving LRC and long-term survivals of localized NKTCL, and meanwhile, deep remission from induction CT also contributed to the survival benefit. Conclusively, we demonstrated that initial CR from induction CT was associated with improved survivals in the modality of CRT for localized NKTCL. These results were consistent with Jing Yang’s study [42]. Yang reported in a study of 193 stage I-II NKTCL patients who received sequential or sandwich chemoradiation. The CR rate of induction GELOX and EPOCHL (etoposide, vincristine, doxorubicin, prednisone, cyclophosphamide, L-asparaginase/pegaspargase) were 75% and 56%, respectively; patients who achieved CR from induction chemotherapy had superior PFS than those with non-CRs (*P* = 0.033) [42].

Actually, early deep remission from upfront or early RT has been reported essential in improving survivals for localized NKTCL and no more than 3 cycles of induction CT prior to RT were recommended [15, 29, 40, 43]. In a multicenter comprehensive study of 1273 localized NKTCL, early RT+CT yielded superior 5-year OS to initial CT +RT (72.2% vs. 58.3%, *P* = 0.017) [14]. In our prior comparison analysis of 75 patients with localized NKTCL receiving first-line IMRT and GDP, upfront RT achieved a higher initial CR rate (91.1% vs. 40.0%; *P* < 0.001), 5-year LRC (90.8% vs. 66.9%; *P* = 0.020) and PFS (81.6% vs. 56.0%, *P* = 0.017), as compared to initial CT followed by RT [29]. In the present study, majority (87.2%) of patients received 2–3 cycles of induction CT prior to RT. In this setting of early RT in our study, initial CR from induction predicted superior survivals over initial non-CRs, indicating that early deep remission from induction CT might also be a positive prognostic factor, and the delay of deep remission might negatively impact survivals for localized NKTCL.

Our results revealed that most treatment failure occurred within the first two years of treatment. In line with published studies, the 5-year cumulative failure rate was 25.4% for localized NKTCL in this study [18, 19, 21, 32, 37]. Furthermore, we demonstrated SF (17.9%) as the main failure pattern for early stage NKTCL receiving first-line CRT. In the old therapy era of CHOP or CHOP-like regimens, the cumulative incidences of SF at 5 years was 24.1% for patients who received CRT compared with 28.5% for those who received RT alone (*P* = 0.758) [32]. Though it was hard to make a direct comparison, the SF rate in this study was slightly lower than that in Li’s study, partly due to the administration of more effective new regimens. However, our results showed SF was still the main failure pattern, especially for those who did not obtain initial CR from induction (30.9%). It was therefore inferred that more intensive systemic therapy was in urgent need to prevent systemic dissemination, which was the key and difficult point for treatment of localized NKTCL in the modern era.

Therefore, we proposed a response-adapted individualized therapy for patients with localized NKTCL in this study. For patients who did not reach CR from induction CT, more intensive therapy of ≥6 total cycles of CT might be recommended (5-year OS, 76.7% vs. 54.7%, *P* = 0.026). However, more intensive therapy did not bring any further survival benefit for patients with initial CR. Conclusively, the induction response might be used for better risk stratification and the induction response-adapted individualized strategy might be of potential value in decision-making in NKTCL. Actually, apart from increased drug dosage which have been discussed above, combination of CRT with new anticancer agents including immune checkpoint inhibitors, epigenetic drugs, adaptive immune therapy and other agents might also be of potential value to decrease SF and prolong survivals for localized NKTCL, some of which are under investigation currently [9, 44–47].

This study has some limitations. Firstly, multiple CT regimens, some of which were not widely used in NKTCL were included in this study. However, all regimens were L-asparaginase/pegaspargase-based in the primary cohort. Besides, though COEPL is modified from CHOP, it is not an ANT-based regimen but incorporates L-asparaginase/pegaspargase. Survival analysis in the present study demonstrated it as an effective regimen for early stage NKTCL. Secondly, though the response assessment techniques were not identical, more than half of patients used PET scanning for induction response assessment. Lastly, this study was limited by its retrospective nature, and the sample size of the validation cohort was comparably small. Therefore, large-sample strictly-designed prospective researches are warrant to validate our conclusion.

## METHODS

### Eligibility and study population

Consecutive patients who were newly-diagnosed localized NKTCL and underwent first-line CT and RT from 2010 to 2020 from Peking University Cancer Hospital (the primary cohort) and Cancer Hospital, Chinese Academy of Medical Sciences and Peking Union Medical College (the validation cohort) were retrospectively reviewed. Inclusion criteria were: (1) newly diagnosed NKTCL with typical morphology and immunophenotype that included CD20/CD79ɑ, CD3ɛ, CD56, TIA-1, Gram-B, perforin, and EBV-encoded RNA *in situ* hybridization, according to WHO classification; (2) tumors primarily occurring in the UADT; (3) Ann Arbor stages I(E)/II(E); (4) patients received first-line induction CT followed by involved-field RT with or without consolidation therapies; (5) at least one measurable lesion; Exclusion criteria were: (1) extra-UADT tumors; (2) advanced (stage III/IV) disease; (3) patients who were treated with initial RT or CT alone; (4) incomplete clinicopathologic and follow-up information.

We observed in clinical practice that a small group of localized NKTCL developed rapidly systemically progression (such as hemophagocytic syndrome) before or in the induction phase, leaving nearly no possibilities for local treatment. It was no doubt that prognosis of these patients was fairly poor. Therefore, to better explore the influence of induction CT response on survivals in the presence of combined RT, we excluded these patients from analyze. Finally, 203 eligible patients who received first-line induction CT and RT were included in the primary cohort and 67 cases in the validation cohort.

### Disease evaluation

Initial clinical evaluation included history and physical examination, PET of the whole body, nasopharyngeal endoscopy of the nasal and oral cavities, magnetic resonance of head and neck, computed tomography of chest, abdomen and pelvis, and bone marrow aspiration.

### Treatment procedure and response assessment

A median 2 (range, 2–6) cycles of induction CT was given to each patient prior to involved-field RT. The CT regimens changed along with time and center. All patients in the primary cohort received L-asparaginase/pegaspargase-based regimens, including COEPL (72.9%), CHOPL/CHOPEL (24.1%), and GDPL/GELOX (3.0%). Patients in the validation cohort received L-asparaginase/pegaspargase-based regimens (42/67, 62.7%) or gemcitabine-based regimens (25/67, 37.3%). Drugs and dosages were listed in Supplementary Table 1. Extended involved-field RT was delivered using intensity modulated radiation (IMRT) or volumetric modulated Arc therapy (VMAT) techniques. The median RT dose was 54 Gy (range, 45-56; dose per fraction, 1.8–2.0 Gy) in the primary cohort and 50 Gy (range, 50–56; dose per fraction, 1.8–2.0 Gy) in the validation cohort. At the completion of RT, additional CT was given to fulfill a total 4–8 cycles of therapy.

Treatment responses were assessed after induction CT (initial response), at the completion of RT with/without consolidation therapy (final response). Examination by PET or computed tomography scans of the head, neck, chest, abdomen, and pelvis; magnetic resonance imaging of the head and neck; were repeated to assess treatment response. Response assessment was followed by the Lugano classification response criteria for NHL [48].

### Statistics

Initial non-response was defined as stable disease (SD) or progression of disease (PD) from induction CT. The ORR was defined as the proportion of patients classified as CR and PR. PFS was defined as the period from the date of treatment until the date of disease progression, relapse, or death from any reason. OS was defined as the interval from the date of first treatment until the date of death from any reason or last follow-up. Survival was analyzed using the Kaplan-Meier method and compared using a log-rank test. Multivariable Cox proportional hazards regression was also performed. All statistical analyses were used SPSS 24.0 software. A two-tailed *P* value *<* 0.05 was considered statistically significant.

## Supplementary Materials

Supplementary Table 1
