# Supplementary material for: Deep remission from induction chemotherapy predicts favorable long-term survivals in early stage extranodal nasal NK/T-cell lymphoma receiving sequential chemotherapy and radiation
Source: Aging (Albany NY). 2022 Nov 1;14(21):8729–44. doi: 10.18632/aging.204355 (PMC9699755; doi:10.18632/aging.204355)
Supplement: Supplementary Table 1 [file aging-14-204355-s001.pdf]

SUPPLEMENTARY TABLE

Supplementary Table 1. Chemotherapy regimens in this study.

Regimen	Drug	Dosage	Period
COEPL	cyclophosphamide	750 mg/m^2 iv d1	q21d
	vincristine	1.4 mg/m^2 iv d1	
	etoposide	80 mg/m^2 iv d1-3	
	prednisone	100 mg/d po d1-5	
	L-asparaginase/pegaspargase	2500 IU/m^2 im d1	
CHOPL/CHOPEL	cyclophosphamide	750 mg/m^2 iv d1	q21d
	doxorubicin	50 mg/m^2 iv d1	
	vincristine	1.4 mg/m^2 (max 2.0 mg) iv d1	
	prednisone	100 mg/d po d1-5	
	-/etoposide	80 mg/m^2 iv d1-3	
	L-asparaginase/pegaspargase	2500 IU/m^2 im d1	
GEMOX/GELOX	Gemcitabine	1000 mg/m^2 iv d1,8	q21d
	oxaliplatin	130 mg/m^2 iv d1	
	-/L-asparaginase/pegaspargase	2500 IU/m^2 im d1	
GDP/GDPL	gemcitabine	1000 mg/m^2 iv d1,8	q21d
	cisplatin	25 mg/m^2 d1-3	
	dexamethasone	20 mg/d po d1-4,11-14	
	-/L-asparaginase/pegaspargase	2500 IU/m^2 im d1	
